# Early decrease of ventilatory ratio after prone position ventilation may predict successful weaning in patients with acute respiratory distress syndrome: A retrospective cohort study

**DOI:** 10.3389/fmed.2022.1057260

**Published:** 2022-12-06

**Authors:** Zhichang Wang, Feiping Xia, Huishui Dai, Hui Chen, Jianfeng Xie, Haibo Qiu, Yi Yang, Fengmei Guo

**Affiliations:** ^1^Jiangsu Provincial Key Laboratory of Critical Care Medicine, Department of Critical Care Medicine, Zhongda Hospital, School of Medicine, Southeast University, Nanjing, China; ^2^Department of Critical Care Medicine, Mingguang People’s Hospital, Chuzhou, China; ^3^Department of Critical Care Medicine, The First Affiliated Hospital of Soochow University, Soochow University, Suzhou, China

**Keywords:** acute respiratory distress syndrome, ventilatory ratio, prone position, risk factors, successful weaning

## Abstract

**Background:**

Previous studies usually identified patients who benefit the most from prone positioning by oxygenation improvement. However, inconsistent results have been reported. Physiologically, pulmonary dead space fraction may be more appropriate in evaluating the prone response. As an easily calculated bedside index, ventilatory ratio (VR) correlates well with pulmonary dead space fraction. Hence, we investigated whether the change in VR after prone positioning is associated with weaning outcomes at day 28 and to identify patients who will benefit the most from prone positioning.

**Materials and methods:**

This retrospective cohort study was performed in a group of mechanically ventilated, non-COVID ARDS patients who received prone positioning in the ICU at Zhongda hospital, Southeast University. The primary outcome was the rate of successful weaning patients at day 28. Arterial blood gas results and corresponding ventilatory parameters on five different time points around the first prone positioning were collected, retrospectively. VR responders were identified by Youden’s index. Competing-risk regression models were used to identify the association between the VR change and liberation from mechanical ventilation at day 28.

**Results:**

One hundred and three ARDS patients receiving prone positioning were included, of whom 53 (51%) successfully weaned from the ventilator at day 28. VR responders were defined as patients showing a decrease in VR of greater than or equal to 0.037 from the baseline to within 4 h after prone. VR responders have significant longer ventilator-free days, higher successful weaning rates and lower mortality compared with non-responders at day 28. And a significant between-group difference exists in the respiratory mechanics improvement after prone (*P* < 0.05). A linear relationship was also found between VR change and compliance of the respiratory system (Crs) change after prone (*r* = 0.32, *P* = 0.025). In the multivariable competing-risk analysis, VR change (sHR 0.57; 95% CI, 0.35–0.92) was independently associated with liberation from mechanical ventilation at day 28.

**Conclusion:**

Ventilatory ratio decreased more significantly within 4 h after prone positioning in patients with successful weaning at day 28. VR change was independently associated with liberation from mechanical ventilation at day 28.

## Background

Prone position ventilation could improve oxygenation while achieving a more homogenous distribution of stress and strain, thus reducing ventilator-induced lung injury (1, 2). After demonstration of significant survival benefits in severe ARDS patients by the PROSEVA study, prone positioning has become one of the most recommended standard managements in moderate to severe ARDS (3, 4). However, not all ARDS patients undergoing prone ventilation could benefit from it. And the poor response to prone positioning was associated with worsened outcomes (5, 6). Mostly, previous studies defined prone positioning response according to the level of PaO_2_:FiO_2_ ratio improvement after prone, expressed in percentage or specific values (5, 7), and inconsistent results were presented (8). It seems that evaluation of prone response by oxygenation improvement may have some limitations.

Prone position ventilation could improve recruitment potential, promote the opening of the collapsed alveoli and reduce alveolar overdistension, hence achieving more homogenous aeration (9), which was deemed as the primary mechanism of alleviating ventilator-induced lung injury. Nevertheless, oxygenation could improve independently of lung recruitment (10). In this way, ventilation monitoring may have better performance in assessing prone responsiveness. Previously, a physiological study proposed that dead space may be more appropriate in evaluating the prone response (11). Furthermore, pulmonary dead space fraction is known to be a more reliable variable that retains its prognostic value over time (12). But the lack of convenience limits its clinical application.

In 2009 Sinha et al. proposed evaluating the physiological dead space fraction by using a rearranged alveolar gas equation for PaCO_2_ without any expired CO_2_ measurement, called the ventilatory ratio (VR) (13). An advantage of VR is that it can be easily calculated using routine bedside variables. And it was demonstrated that VR correlates well with pulmonary dead space in ARDS and may be used as a simple bedside index to monitor impaired ventilation in ARDS (14). However, its prognostic utility following prone positioning is unclear.

In this context, we investigated whether the change in VR after prone positioning was associated with weaning outcomes at day 28, aimed to identify patients who will benefit the most from prone positioning.

## Materials and methods

### Study design and patients

We conducted a retrospective cohort study of adult ARDS patients (age ≥ 18 years) who received prone positioning in a 60-bed general intensive care unit (ICU) in Zhongda Hospital, Southeast University, China, between January 1, 2017, and March 31, 2022. No COVID-19 patients were admitted to our hospital during the study period according to the local health policies during COVID-19 pandemic. While mechanically ventilated, moderate to severe ARDS patients who received at least one prone session were included. Then we excluded patients who received prone positioning for less than 12 h at the first prone session and patients who received V-V ECMO (venovenous extracorporeal membrane oxygenation) therapy during the first prone session. All patients fulfilled the Berlin definition of moderate to severe ARDS before proning (15). The decision to initiate a prone position and the timing were at the discretion of attending doctors. To protect individual privacy, anonymized and deidentified information was analyzed. The Institutional Review Board (IRB) of Zhongda Hospital waived the requirement for written informed consent and approved this study (approval number 2022ZDSYLL268-P01).

### Study outcomes and variables

We focused on patient ventilator liberation to explore the relationship between VR change during the first prone session and patient outcomes (i.e., VR response to the first prone session and patient outcomes). The main outcome was the rate of successful weaning at 28-day, which was defined as a lack of invasive or non-invasive ventilation for more than 48 h after extubation for endotracheally intubated patients and no ventilator assistance for more than 48 h for tracheostomized patients. Patients not weaned from mechanical ventilation until day 28 or who died before successful weaning were defined as weaning failure. Time to successful weaning and time to death were also collected for the following regression analysis, which was defined as time from initiation of the first prone session to event occurred (i.e., successful weaning or death).

Twenty eight-day mortality and ventilator-free days in 28 days were also collected for comparison between VR responders and non-responders (detailed definitions of the responders and non-responders were shown in Section “Statistical analysis”). Ventilator-free days were defined as the number of days alive and free from mechanical ventilation for at least 48 consecutive hours (16).

Data of arterial blood gas analysis and corresponding ventilatory parameters were collected retrospectively from clinical documentation. Time points include before the first prone session (time baseline), within 4 h after the patient was turned to the prone position (time P1), from 4 to 12 h after prone positioning (time P2), 12 h after prone positioning to the end of prone positioning (time P3) and within 4 h after the patient was returned to the supine position (time S1). Only arterial blood gas result corresponding to the best oxygenation (i.e., PaO_2_:FiO_2_ ratio) remained in the final analysis if the patient had multiple arterial blood gas results at the same time interval. Ventilatory parameters include tidal volume (Vt), respiratory rate (RR), and positive end-expiratory pressure (PEEP) were extracted at the same time points as the arterial blood gas result. We derived values for minute ventilation, tidal volume per predicted body weight, ventilatory ratio, and corrected minute volumes.

Ventilatory ratio (VR) was defined as [minute ventilation (mL/min) * PaCO2 (mm Hg)/[predicted body weight * 100 (mL/min) * 37.5 (mm Hg)]] (13, 14). ΔVR was defined as VR at specific time point minus VR at baseline (e.g., ΔVR at time P1 = VR at time P1-VR at baseline). ΔPaO_2_:FiO_2_ was calculated the same way as ΔVR. Minute ventilation (V_*E*_) was defined as (tidal volume [ml] * respiratory rate [times/min]/1,000 [ml/L]). The driving pressure was defined as the difference between plateau pressure and PEEP_*tot*_, which were measured by inspiratory/expiratory hold maneuver. And the respiratory system compliance (C_*rs*_) was calculated as (tidal volume [ml]/driving pressure [cmH_2_O]).

### Statistical analysis

Categorical variables are expressed as frequencies with percentages, and continuous variables are expressed as mean with standard deviation (SD) or median with interquartile range (IQR). Normally distributed quantitative variables were compared using the Student *t*-test, and non-normally distributed quantitative variables were compared using the Mann-Whitney U test. Qualitative variables were compared using the chi-square test. A generalized estimating equation (GEE) was used to compare differences in VR and the change of VR from the baseline to during the first prone session. Data were assumed to be missing at random with no imputation or interpolation of missing values employed.

We generated receiver operating characteristic (ROC) curves and estimated the area under the curve (AUROC) to determine the predictive value of change in VR and PaO_2_:FiO_2_ ratio between the baseline and during the first prone session for successful weaning from mechanical ventilation at 28-day. Best prediction value among the four time points was identified. Then, the optimal cutoff values were determined based on Youden’s index, which maximizes the sum of sensitivity and specificity, responders or non-responders were discriminated afterward. Linear regression model was used to investigate the correlation between the VR change and C_rs_ change, together with the PaO_2_:FiO_2_ change and C_*rs*_ change. Liberation from ventilator was analysed with death as a competing event using a Fine and Gray with proportional hazard model. VR change within 4 h after prone positioning (ΔVR at time P1) and PaO_2_:FiO_2_ change within 4 h after the patient was turned to supine position (ΔPF at time S1) were entered into the multivariable model as continuous variables. Multivariable regression model was adjusted by age, SOFA score, and time from intubation to the first prone session. The results are presented as subdistribution hazard ratio (sHR) with 95% CI. All analyses were performed blinded to participant identifying information. All analyses were two-tailed, and *p*-values of less than 0.05 were considered significant. STATA (Version 17.0 for Windows; Stata Corp., College Station, TX, United States) was used for all statistical analyses.

## Results

### Patients recruitment and baseline characteristics

**FIGURE 1:**
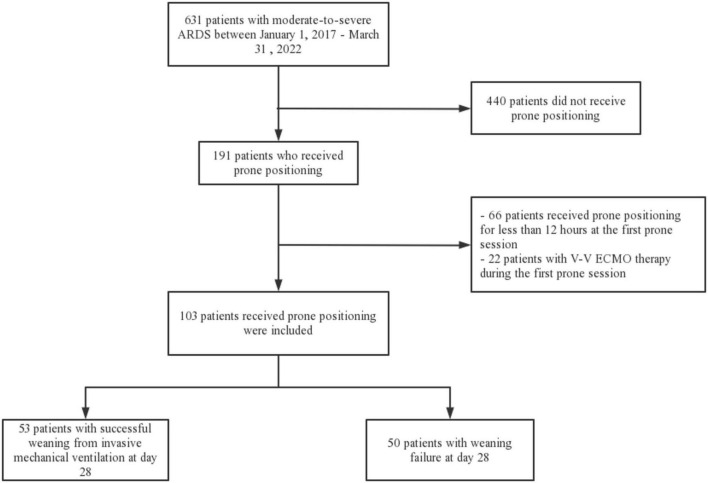
Flowchart of the study population. ARDS, acute respiratory distress syndrome; V-V ECMO, venovenous extracorporeal membrane oxygenation.

Six hundred and thirty one patients with moderate-to-severe ARDS were identified during the study period, and 191 patients received prone positioning. Sixty-six patients who received prone positioning for less than 12 h at the first prone session and twenty-two patients who received V-V ECMO therapy during the first prone session were excluded (Figure 1). Of the 103 patients included, 53 patients weaned successfully from the ventilator at 28-day, the overall successful weaning rate was 51%.

Characteristics of the patients were shown in Table 1, grouped by weaning status. The leading cause of ARDS was pneumonia (73.8%), and the PaO_2_:FiO_2_ ratio before prone mostly ranged from 100 to 150 mmHg (49.5%). More than half of the patients were diagnosed with hypertension (47.6%) or diabetes (10.7%). Age, ARDS severity, ARDS etiology, Sequential Organ Failure Assessment (SOFA) scores and Acute Physiology and Chronic Health Evaluation (APACHE) II scores did not differ significantly between the two groups.

**TABLE 1:** Characteristics of the patients at baseline.

Characteristic	Total (*n* = 103)	Successful weaning (*n* = 53)	Weaning failure (*n* = 50)	*P*
Age, years	69 ± 14	66 ± 14	73 ± 14	0.026
Gender, Male	85 (82.5)	43 (81.1)	42 (84.0)	0.702
Body-mass index, kg/m^2^	23.2 ± 3.5	23.3 ± 3.7	23.0 ± 3.3	0.592
APACHE II score	21.6 ± 7.2	20.4 ± 6.5	22.8 ± 7.7	0.084
SOFA score	8.3 ± 3.1	7.8 ± 3.0	8.8 ± 3.1	0.100
**ARDS severity**				0.246
150 < PaO_2_:FiO_2_ < 200	30 (29.1)	18 (34.0)	12 (24.0)	
100 < PaO_2_:FiO_2_ < 150	51 (49.5)	22 (41.5)	29 (58.0)	
PaO_2_:FiO_2_ < 100	22 (21.4)	13 (24.5)	9 (18.0)	
**Primary cause of ARDS**				0.487
Pneumonia	76 (73.8)	37 (69.8)	39 (78.0)	
Aspiration	10 (9.7)	5 (9.4)	5 (10.0)	
Non-pulmonary sepsis	17 (16.5)	11 (20.8)	6 (12.0)	
**Comorbidities**				
Diabetes	11 (10.7)	4 (7.6)	7 (14.0)	0.289
Hypertension	49 (47.6)	30 (56.6)	19 (38.0)	0.059
Chronic liver disease	6 (5.8)	4 (7.6)	2 (4.0)	0.442
Chronic kidney disease	9 (7.2)	5 (8.3)	4 (6.2)	0.797
Coronary artery disease	22 (21.4)	11 (20.8)	11 (22.0)	0.878
COPD	4 (3.9)	1 (1.9)	3 (6.0)	0.280
Median time from intubation to prone, days	1.1 (0.3–2.4)	0.8 (0.2–1.8)	1.9 (0.4–4.2)	0.006*
**Pre-prone parameters**				
Tidal volume, ml/kg PBW	6.5 ± 1.8	7.1 ± 1.7	6.7 ± 1.5	0.204
Respiratory rate, breaths/min	21 ± 6	21 ± 6	22 ± 6	0.122
PEEP, cm H_2_O	10 ± 3	10 ± 3	9 ± 3	0.439
§Driving pressure, cm H_2_O	12 ± 4	11 ± 3	12 ± 4	0.056
§Plateau pressure, cm H_2_O	21 ± 5	21 ± 5	22 ± 5	0.469
FiO_2_, %	61 ± 20	61 ± 21	62 ± 19	0.946
Minute ventilation (V_*E*_), L/min	9.2 ± 3.3	9.0 ± 3.4	9.4 ± 3.1	0.533
§Compliance, ml/cm H_2_O	36 ± 13	39 ± 14	32 ± 12	0.052
**Pre-prone blood gases**				
pH	7.38 ± 0.07	7.38 ± 0.07	7.38 ± 0.07	0.998
PaCO_2_, mm Hg	43 ± 10	41 ± 10	44 ± 11	0.115
V_*E*_:PaCO_2_, ml⋅min^–1^⋅mm Hg^–1^	233 ± 116	237 ± 128	229 ± 104	0.723
PaO_2_, mm Hg	77 ± 19	75 ± 17	79 ± 20	0.272
PaO_2_:FiO_2_, mmHg	133 ± 35	132 ± 40	134 ± 30	0.834
Bicarbonate, mmol/L	25 ± 5	24 ± 5	26 ± 6	0.092
Lactate, mmol/L	1.9 ± 1.0	1.8 ± 1.1	1.9 ± 1.0	0.617
Ventilatory ratio	1.62 ± 0.56	1.54 ± 0.55	1.70 ± 0.55	0.159

Data are reported as *n* (%), mean ± SD, or median (1st–3rd quartile). *p < 0.05; §Baseline respiratory mechanics data were partly missed at baseline, available data distribution was *n* = 70 for total, *n* = 36 for successful weaning group and *n* = 34 for weaning failure group. Missing percentage was 25.3% for total, 32.1% for successful weaning group, 32.0% for weaning failure group. ARDS, acute respiratory distress syndrome; APACHE, acute physiology and chronic health evaluation; SOFA, sequential organ failure assessment score; COPD, chronic obstructive pulmonary disease; PEEP, positive end-expiratory pressure.

The results of arterial blood gas analysis, respiratory system mechanics and ventilator settings at baseline did not differ significantly between the two groups (Table 1). Weaning failure patients were like to have higher bicarbonate, lower tidal volume and compliance at baseline. At baseline, the mean VR was 1.62 ± 0.56, mean PaO_2_:FiO_2_ ratio level was 133 ± 35 mmHg.

### Prone positioning

The median time from intubation to the first prone session was shorter for successful weaning patients compared with weaning failure patients (0.8 days, IQR [0.2–1.8] vs. 1.9 days, IQR [0.4–4.2]; *P* = 0.006). During the initial prone session adjunctive therapies did not differ significantly between groups, although the proportion tend to be higher in weaning failure patients (Table 2). For all patients, the median duration of the first prone session was 16.0 h (14.2–17.2 h), the median number of prone sessions was 4 (3–7), the median period of proning was 5 days (4–8 days), and the total prone duration was 62.8 h (42.3–107.7 h). Weaning failure patients tend to receive more sessions and longer time of prone positioning, although statistically non-significant.

**TABLE 2:** Characteristics of adjunctive therapies and prone sessions.

Variables	Total (*n* = 103)	Successful weaning (*n* = 53)	Weaning failure (*n* = 50)	*P*
Neuromuscular blockers	29 (28.2)	11 (20.8)	18 (36.0)	0.086
Vasopressors	12 (11.7)	3 (5.7)	9 (18.0)	0.051
Renal replacement therapy	14 (13.6)	4 (7.6)	10 (20.0)	0.065
Duration of the first prone session, h	16.0 (14.2–17.2)	15.6 (14.1–16.9)	16.3 (14.2–17.6)	0.118
Prone sessions	4 (3–7)	4 (3–6)	5 (2–8)	0.790
Period of proning, days	5 (4–8)	5 (4–7)	6 (3–9)	0.661
Total prone duration, h	62.8 (42.3–107.7)	59.2 (46.4–84.6)	83.4 (32.6–125.4)	0.398

Data are reported as *n* (%), or median (1st–3rd quartile).

### Ventilatory ratio response

**FIGURE 2:**
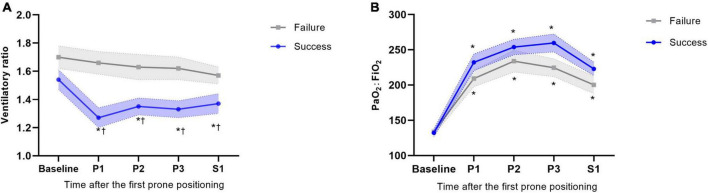
Physiological response to prone positioning according to subgroups of successful weaning and weaning failure at day 28. **(A)** Ventilatory ratio response to prone positioning according to different subgroups. **(B)** PaO_2_:FiO_2_ response to prone positioning according to different subgroups. The error interval between dotted line indicate the SE. *p < 0.05 of absolute values of different time points vs. baseline within group; ^†^*p* < 0.05 of absolute value of success group vs. failure group at the same time point.

**FIGURE 3:**
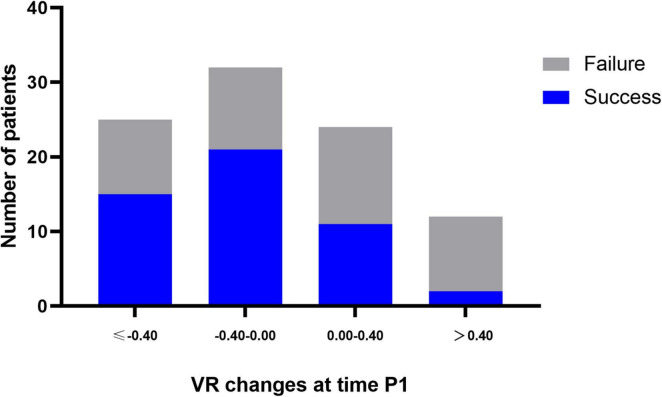
Proportion of patients with successful weaning according to the change of VR within 4 h after prone positioning (time P1). Blue plots represent successful weaning patients at day 28, whereas, gray plots represent weaning failure patients at day 28. –0.40 was 33rd percentile of VR change at time P1.

Among 53 successfully weaned patients, the VR decreased from a mean of 1.54 ± 0.55 at baseline to 1.27 ± 0.48 at time P1. The results observed at times P2, P3, and S1 were almost constant and similar to that observed at time P1. Among 50 weaning failure patients, the VR did not change markedly from baseline to time S1. The between-group differences were significant at all time points through the first prone period (Figure 2A). Changes in the VR were significantly different between groups at time P1 (Mean difference 0.23, 95% CI 0.02–0.48; *P* = 0.025) (Supplementary Table 1). Furthermore, the proportion of patients with successful weaning was 60.0% when VR decreased by more than 0.40 from baseline to time P1; in contrast, for patients VR increased by more than 0.40, the proportion of successful weaning was only 21.4% (Figure 3).

For oxygenation response, among 53 successfully weaning patients, the PaO_2_:FiO_2_ ratio increased from a mean of 132 ± 39 mmHg at baseline to 260 ± 68 mmHg at time P3. After resupine, the PaO_2_:FiO_2_ ratio decreased to a mean of 223 ± 64 mmHg. No between-group difference was observed through the first prone period (Figure 2B). Changes in PaO_2_:FiO_2_ ratio were found significantly different between groups at time P3 (Mean difference 36.64, 95% CI 2.53–70.76; *P* = 0.035) (Supplementary Table 1). The values for other blood gas results and mechanical ventilation parameters are shown in Supplementary Table 1.

### Prediction of weaning by ventilatory ratio response

During the first prone positioning, the AUROC value of the changes in the VR between baseline and time P1 was significant for predicting the probability of successful weaning at day 28 (AUROC 0.64, 95% CI 0.53–0.75; *P* = 0.015) (Supplementary Table 2 and Supplementary Figure 1). The optimal cut-off value for the changes of the VR between baseline and time P1 was –0.037, with a sensitivity of 55.6% and specificity of 70.0%. “VR responders” were defined as patients showing a decrease in the ventilatory ratio of greater than or equal to 0.037 from the baseline to time P1.

**FIGURE 4:**
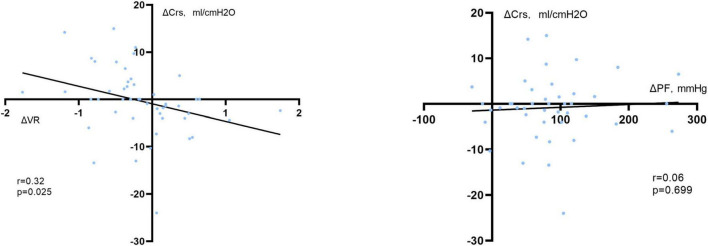
Correlation between changes in the static compliance of the respiratory system (ΔCrs) and changes in VR at time P1 (ΔVR, **left**) or PaO_2_:FiO_2_ ratio at time S1 (ΔPF, **right**) when compared with the supine position. VR was calculated as a ratio which compares actual measurements and predicted values of minute ventilation and PaCO_2_ as proposed previously. Crs was calculated as Vt divided by driving pressure. And driving pressure was acquired during an end-inspiration and end-expiration maneuver, calculated as plateau pressure minus end-expiratory pressure.

Moreover, changes in static respiratory system compliance and driving pressure were statistically significant between VR responders and non-responders (Supplementary Table 4). A linear relationship was also found between the changes in VR from baseline to time P1 and the changes in respiratory system compliance (Crs) from baseline to during prone positioning (*r* = 0.32, *P* = 0.025) (Figure 4). However, such relationships were not found in PaO_2_:FiO_2_ ratio. A detailed description of the predictive value of the changes of PaO_2_:FiO_2_ ratio and its relationship with respiratory mechanics are shown in Supplementary Tables 2, 5.

### Clinical outcomes and predictors of liberation from mechanical ventilation

In our cohort, overall ventilator-free days was 2.2 [0.0–7.0] days, mortality was 41%. For VR responders, as compared to the non-responders group, the number of ventilator-free days was significantly higher [4.1 (0.0–7.1) vs. 0.0 (0.0–7.0) days, *P* = 0.033] and mortality tend to be lower (36.4 vs. 52.5%, *P* = 0.117) (Supplementary Table 3).

The multivariable competing-risk analysis (Table 3) showed that VR change within 4 h after prone positioning (sHR 0.57, 95% CI 0.35–0.92; *p* = 0.022) and SOFA score (sHR 0.91, 95% CI 0.83–0.99; *p* = 0.045) was independently associated with liberation from mechanical ventilation at 28 days after adjustment for covariates, while PaO_2_:FiO_2_ change was not.

**TABLE 3:** Competing-risk regression model for predicting liberation from mechanical ventilation at day 28.

Variable (reference)	Univariate analysis		Multivariate analysis	
	sHR (95% CI)	*P*	sHR (95% CI)	*P*
ΔVR at time P1	0.64 (0.41–1.01)	0.057	0.57 (0.35–0.92)	**0.022**
ΔPF at time S1, mmHg	1.00 (1.00–1.01)	0.072	1.00 (1.00–1.01)	0.234
Gender (male)	0.98 (0.96–1.00)	0.019		–
Age, years	1.02 (0.95–1.09)	0.605	0.98 (0.97–1.00)	0.059
Body-mass index, kg/m^2^	0.97 (0.93–1.00)	0.069		–
SOFA score	0.94 (0.86–1.01)	0.103	0.91 (0.83–0.99)	**0.045**
APACHE II score	1.12 (0.95–1.33)	0.187		–
Baseline TV, ml/kg PBW	1.02 (0.94–1.11)	0.607		–
Baseline PEEP, cm H_2_O	1.00 (0.99–1.01)	0.835		–
Baseline PF, mmHg	0.56 (0.29–1.06)	0.075		–
Neuromuscular blockers (No)	0.37 (0.11–1.20)	0.098		–
Vasopressors (No)	0.45 (0.15–1.31)	0.144		–
Renal replacement therapy (No)	0.81 (0.68–0.96)	0.017		–
Median time from intubation to the first prone session, days	0.96 (0.91–1.01)	0.114	0.79 (0.61–1.01)	0.062
Biocarbonate, mmol/L	0.96 (0.92–1.00)	0.072		–

Significant *P*-values are highlighted in bold. VR, ventilatory ratio; PF, PaO_2_:FiO_2_; SOFA, sequential organ failure assessment; APACHE, acute physiology and chronic health evaluation; TV, tidal volume; ARDS, acute respiratory distress syndrome; PEEP, positive end-expiratory pressure; CI, confidence interval; sHR, subdistribution hazard ratio.

## Discussion

To our knowledge, it’s the first time we explore the VR, a simple bedside method, its change during the first prone session, and the association with liberation from mechanical ventilation in moderate to severe ARDS patients. The main results of the study could be summarized as follows. (1) The VR during the first prone session differed significantly between successful weaning patients and weaning failure patients at day 28. (2) The change in VR from baseline to within 4 h after prone positioning may predict liberation from mechanical ventilation at day 28 and have a linear relationship with static respiratory system compliance changes. (3) VR change within 4 h after prone positioning was independently associated with liberation from mechanical ventilation at 28 days even after controlling for other prognostic variables.

The VR was proposed by Sinha in 2009, calculated as a ratio that compares actual measurements and predicted values of minute ventilation and PaCO_2_ (13). It was deemed to reflect the combined effect of dead space and shunt on CO_2_ elimination, especially in diseases such as ARDS, where there is likely to be a massive ventilation-perfusion mismatch. In 2019, a physiological analysis demonstrated that VR correlates well with dead space fraction in ARDS patients, and higher values are associated with increased risk of adverse outcomes, making VR promising as a simple bedside index evaluating impaired ventilation in ARDS (14). Prior study examined the VR change following prone position maneuver and found an improvement in VR after the initial prone session in ARDS patients. Still, scarce studies explore the prognostic utility of VR following prone position maneuver (7). In this study, we found a significant difference in VR at all time points through the initial prone period between successfully weaning patients and weaning failure patients. And there is an upward trend in successful weaning proportion following the degree of VR improvement at an early stage after initial prone (within 4 h). After multivariable regression analysis, our results showed that VR change within 4 h after prone positioning was independently associated with liberation from mechanical ventilation at 28 days even after controlling for other prognostic variables.

Previous studies examining oxygenation response to prone have demonstrated mixed results. One secondary analysis based on PROSEVA data failed to show any change in the PaO_2_:FiO_2_ ratio that differed between survivors and non-survivors (8). Recently, a retrospective study found that the PaO_2_:FiO_2_ ratio after the first prone positioning differed significantly between ICU survivors and non-survivors, and the percentage of PaO_2_:FiO_2_ ratio change may be a significant predictor of survival in ARDS patients who received prone positioning (5).

Mix results can be partly attributed to the mechanism of prone maneuver-related PaO_2_:FiO_2_ ratio improvement. First, when patients shift from supine to prone, the primary phenomenon is the balance between the release of dorsal atelectasis and its formation in the ventral area. This density redistribution was observed in many early ARDS patients (17, 18). Rossi et al. observed such phenomenon and they demonstrated that the PaO_2_:FiO_2_ ratio changes after prone positioning correlated with the balance between resolution of dorsal atelectasis and formation of ventral atelectasis (19). Second, ventilation/perfusion matching is improved simply due to the decreased hydrostatic gradient after prone or changes of global cardiac output, thus resulting in oxygenation improvement (10, 20). Whereas, the first mechanism is much more critical in terms of enhancing protection from ventilator-induced lung injury. Gattinoni et al. (21) reported that an increase in PaO_2_:FiO_2_ ratio > 20 mmHg after 6 h of prone positioning is not a predictor of the patient’s prognosis, whereas, a decline in PaCO_2_ ≥ 1 mmHg is. In our present study, the VR was used as a tool to monitor ventilation impairment just as PaCO_2_ did, VR responders had a reduced duration of mechanical ventilation at day 28 compared with VR non-responders, and VR change was independently associated with liberation from mechanical ventilation whereas PaO_2_:FiO_2_ change was not. This remind us that bedside ventilatory ratio monitoring may be a more appropriate way when considering ventilator liberation in moderate to severe ARDS patients after prone. Future studies are needed to address this critical issue.

Shifting from supine to prone resulted in a decrease in chest wall compliance, which was caused by a greater rigidity of the rib cage component of the chest wall in the prone position when compared with the supine position (22). The decrease in chest wall compliance may be compensated if dorsal recruitment overcomes ventral derecruitment, which can lead to increased respiratory system compliance. In our present study, 29 of 43 patients were PaO_2_:FiO_2_ responders (increased PaO_2_:FiO_2_ ratio > 22.95 mmHg within 4 h after resupine). However, changes in Pplat, Crs, and driving pressure did not differ between PaO_2_:FiO_2_ responders and PaO_2_:FiO_2_ non-responders. In contrast, 29 of 49 patients were VR responders (decreased VR > 0.037 within 4 h after PP). VR responders had a significant improvement in Crs, and driving pressure, compared with non-responders. And a linear relationship was found between the changes in VR at time P1 and the changes in static respiratory system compliance from baseline to during prone positioning. Similar findings were also reported by Charron et al. (11) that dead space fraction, and Crs were significantly more altered in PaCO_2_ responders than non-responders when compared with the PaO_2_:FiO_2_ classification. One prospective cohort study reported that improvement in respiratory system compliance contributes to the recovery of pulmonary function (23). Accordingly, these findings suggest that improvement in respiratory system compliance during prone positioning may have contributed to better outcomes in patients receiving prone position ventilation, and it seems that dynamic evaluation of ventilatory impairment was more relevant to respiratory system compliance change during prone; hence, reflect the recovery of pulmonary function in ARDS.

Another important finding in our present study was that the difference in the VR was found at within 4 h after the first prone session between successful weaning patients and weaning failure patients at day 28, which is a relatively earlier stage after prone positioning. While the difference in PaO_2_:FiO_2_ ratio was found after 12 h from the initiation of the first prone positioning. Similar findings were also reported by Scaramuzzo et al. that sustained oxygenation improvement after returning to supine position was associated with early liberation from mechanical ventilation among prone patients (6). Which may suggested that the VR improvement manifest at an early stage after prone in successful weaning patients, while PaO_2_:FiO_2_ ratio improvement persistent for a long time indicates successful weaning from ventilator. Owing to the missing data and retrospective study characteristic of our study, such results should be interpreted carefully, and future prospective study is required.

Our results may potentially help clinicians in identifying patients who may get the best out of prone positioning: those with significant decreasing in VR at early stage. Hence, other interventions, such as early application of ECMO may be considered in patients who present reversely. Recently, a prolonged time of prone positioning was suggested in order to maintain oxygenation improvement (24). Similar tendency was observed in our population when more prone sessions and longer total prone time were applied to non-responder patients, but significant difference still presented in 28-day outcomes between VR responders and non-responders. It is worth to explore in future studies if prolonged prone session could reverse patients’ poor response to prone and who could still benefit from prone position ventilation or responsiveness to prone was inherently determined.

Our study had several limitations. First, it’s retrospective, single center study, and data missing or inadequate data may have affected the outcomes. Another limitation is that 29.1% of the patients included in our study have a baseline PaO_2_:FiO_2_ ratio between 150 and 200 mmHg, while previous guideline recommended PaO_2_:FiO_2_ ratio lower than 150 mmHg as an indication for prone positioning. Despite this, we just analyzed the response to the first prone session. Further studies should evaluate the response to subsequent prone sessions and its relationship with patient outcomes.

## Conclusion

Ventilatory ratio decreased more significantly within 4 h after prone positioning in patients with successfully weaning at day 28. The respiratory response to prone positioning appeared more relevant when VR rather than the PaO_2_:FiO_2_ ratio was used. VR change within 4 h after prone positioning was independently associated with liberation from mechanical ventilation at day 28.

## Data availability statement

The raw data supporting the conclusions of this article will be made available by the authors, without undue reservation.

## Ethics statement

The Institutional Review Board (IRB) of Zhongda Hospital waived the requirement for written informed consent and approved this study (approval number: 2022ZDSYLL268-P01).

## Author contributions

ZW, FX, FG, HQ, and YY designed the study. ZW, FX, HD, HC, and JX acquired, analyzed, and interpreted the data. ZW drafted the manuscript. FX, HC, JX, FG, HQ, and YY made critical revision of the manuscript. All authors contributed to the article and approved the submitted version.

## References

[1] Broccard A Shapiro RS Schmitz LL Adams AB Nahum A Marini JJ. Prone positioning attenuates and redistributes ventilator-induced lung injury in dogs. *Crit Care Med.* (2000) 28:295–303. 10.1097/00003246-200002000-00001 10708156

[2] Perchiazzi G Rylander C Vena A Derosa S Polieri D Fiore T Lung regional stress and strain as a function of posture and ventilatory mode. *J Appl Physiol.* (1985) 2011:1374–83. 10.1152/japplphysiol.00439.2010 21393463

[3] Aoyama H Uchida K Aoyama K Pechlivanoglou P Englesakis M Yamada Y Assessment of therapeutic interventions and lung protective ventilation in patients with moderate to severe acute respiratory distress syndrome: a systematic review and network meta-analysis. *JAMA Netw Open.* (2019) 2:e198116. 10.1001/jamanetworkopen.2019.8116 31365111 PMC6669780

[4] Guérin C Reignier J Richard J-C Beuret P Gacouin A Boulain T Prone positioning in severe acute respiratory distress syndrome. *N Engl J Med.* (2013) 368:2159–68. 10.1056/NEJMoa1214103 23688302

[5] Lee HY Cho J Kwak N Choi SM Lee J Park YS Improved oxygenation after prone positioning may be a predictor of survival in patients with acute respiratory distress syndrome. *Crit Care Med.* (2020) 48:1729–36. 10.1097/CCM.0000000000004611 33003079

[6] Scaramuzzo G Gamberini L Tonetti T Zani G Ottaviani I Mazzoli CA Sustained oxygenation improvement after first prone positioning is associated with liberation from mechanical ventilation and mortality in critically ill COVID-19 patients: a cohort study. *Ann Intensive Care.* (2021) 11:63. 10.1186/s13613-021-00853-1 33900484 PMC8072095

[7] Camporota L Sanderson B Chiumello D Terzi N Argaud L Rimmelé T Prone position in COVID-19 and – COVID-19 acute respiratory distress syndrome: an international multicenter observational comparative study. *Crit Care Med.* (2022) 50:633–43. 10.1097/CCM.0000000000005354 34582426 PMC8923275

[8] Albert RK Keniston A Baboi L Ayzac L Guérin C. Prone position-induced improvement in gas exchange does not predict improved survival in the acute respiratory distress syndrome. *Am J Respir Crit Care Med.* (2014) 189:494–6. 10.1164/rccm.201311-2056LE 24528322

[9] Guérin C Albert RK Beitler J Gattinoni L Jaber S Marini JJ Prone position in ARDS patients: why, when, how and for whom. *Intensive Care Med.* (2020) 46:2385–96. 10.1007/s00134-020-06306-w 33169218 PMC7652705

[10] Dantzker DR Lynch JP Weg JG. Depression of cardiac output is a mechanism of shunt reduction in the therapy of acute respiratory failure. *Chest.* (1980) 77:636–42. 10.1378/chest.77.5.636 6988180

[11] Charron C Repesse X Bouferrache K Bodson L Castro S Page B PaCO2 and alveolar dead space are more relevant than PaO2/FiO2 ratio in monitoring the respiratory response to prone position in ARDS patients: a physiological study. *Crit Care.* (2011) 15:R175. 10.1186/cc10324 21791044 PMC3387618

[12] Raurich JM Vilar M Colomar A Ibáñez J Ayestarán I Pérez-Bárcena J Prognostic value of the pulmonary dead-space fraction during the early and intermediate phases of acute respiratory distress syndrome. *Respir Care.* (2010) 55:282–7. 20196876

[13] Sinha P Fauvel NJ Singh S Soni N. Ventilatory ratio: a simple bedside measure of ventilation. *Br J Anaesth.* (2009) 102:692–7. 10.1093/bja/aep054 19346233

[14] Sinha P Calfee CS Beitler JR Soni N Ho K Matthay MA Physiologic analysis and clinical performance of the ventilatory ratio in acute respiratory distress syndrome. *Am J Respir Crit Care Med.* (2019) 199:333–41. 10.1164/rccm.201804-0692OC 30211618 PMC6363976

[15] Ranieri VM Rubenfeld GD Thompson BT Ferguson ND Caldwell E Fan E Acute respiratory distress syndrome: the Berlin Definition. *JAMA.* (2012) 307:2526–33. 10.1001/jama.2012.5669 22797452

[16] Béduneau G Pham T Schortgen F Piquilloud L Zogheib E Jonas M Epidemiology of weaning outcome according to a new definition. the WIND study. *Am J Respir Crit Care Med.* (2017) 195:772–83. 10.1164/rccm.201602-0320OC 27626706

[17] Gattinoni L Pesenti A Carlesso E. Body position changes redistribute lung computed-tomographic density in patients with acute respiratory failure: impact and clinical fallout through the following 20 years. *Intensive Care Med.* (2013) 39:1909–15. 10.1007/s00134-013-3066-x 24026295

[18] Cornejo RA Díaz JC Tobar EA Bruhn AR Ramos CA González RA Effects of prone positioning on lung protection in patients with acute respiratory distress syndrome. *Am J Respir Crit Care Med.* (2013) 188:440–8. 10.1164/rccm.201207-1279OC 23348974

[19] Rossi S Palumbo MM Sverzellati N Busana M Malchiodi L Bresciani P Mechanisms of oxygenation responses to proning and recruitment in COVID-19 pneumonia. *Intens Care Med.* (2022) 48:56–66. 10.1007/s00134-021-06562-4 34825929 PMC8617364

[20] Richter T Bellani G Scott Harris R Vidal Melo MF Winkler T Venegas JG Effect of prone position on regional shunt, aeration, and perfusion in experimental acute lung injury. *Am J Respir Crit Care Med.* (2005) 172:480–7. 10.1164/rccm.200501-004OC 15901611 PMC2718529

[21] Gattinoni L Vagginelli F Carlesso E Taccone P Conte V Chiumello D Decrease in PaCO2 with prone position is predictive of improved outcome in acute respiratory distress syndrome. *Crit Care Med.* (2003) 31:2727–33. 10.1097/01.CCM.0000098032.34052.F9 14668608

[22] Pelosi P Tubiolo D Mascheroni D Vicardi P Crotti S Valenza F Effects of the prone position on respiratory mechanics and gas exchange during acute lung injury. *Am J Respir Crit Care Med.* (1998) 157:387–93. 10.1164/ajrccm.157.2.97-04023 9476848

[23] Schmidt M Pham T Arcadipane A Agerstrand C Ohshimo S Pellegrino V Mechanical ventilation management during extracorporeal membrane oxygenation for acute respiratory distress syndrome. an international multicenter prospective cohort. *Am J Respir Crit Care Med.* (2019) 200:1002–12. 10.1164/rccm.201806-1094OC 31144997

[24] Carsetti A Damia Paciarini A Marini B Pantanetti S Adrario E Donati A. Prolonged prone position ventilation for SARS-CoV-2 patients is feasible and effective. *Crit Care.* (2020) 24:225. 10.1186/s13054-020-02956-w 32414420 PMC7226707

